# The β-polymorph of uranium phosphide selenide

**DOI:** 10.1107/S1600536811049592

**Published:** 2011-11-30

**Authors:** George N. Oh, James A. Ibers

**Affiliations:** a Department of Chemistry, Northwestern University, 2145 Sheridan Rd., Evanston, IL 60208-3113, USA

## Abstract

β-UPSe was synthesized from the reaction of U_2_Se_3_, P and Se in a CsCl flux in a fused-silica tube. It crystallizes with four formula units in the tetra­gonal space group *I*4/*mmm* in the UGeTe structure type. The asymmetric unit comprises one U (site symmetry 4*mm*), one Se (4*mm*), and one P (*mmm*.) atom. The U atom is coordinated in a monocapped square-anti­prismatic arrangement, where the square face is formed by P atoms and the other five vertices are Se atoms. The P site is disordered about a mirror plane, showing half-ocupancy for each of the two resulting P atoms. The title structure is related to that of α-UPSe, which crystallizes with two formula units in the tetra­gonal space group *P*4/*nmm* in the PbFCl structure type.

## Related literature

Whereas β-UPSe crystallizes in the UGeTe structure type (Haneveld & Jellinek, 1969 ▶), α-UPSe (Hulliger, 1968 ▶; Zygmunt *et al.*, 1974*a* ▶) crystallizes in the PbFCl structure type (Nieuwenkamp & Bijvoet, 1932 ▶). Isostructural compounds U*TQ* have been synthesized (*T* = P–Bi, *Q* = Se–Te) (Hulliger, 1968 ▶; Leciejewicz & Zygmunt, 1972 ▶; Haneveld & Jellinek, 1969 ▶; Zygmunt *et al.*, 1974**a* ▶,b* ▶; Pietraszko & Lukaszewicz, 1975 ▶; Pearson, 1985 ▶). Magnetic studies have been performed on single crystals of β-UPSe synthesized by the vapor-transport method (Kaczorowski *et al.*, 1995 ▶), and other compounds with formula U*TQ* (Troc, 1987 ▶). For synthetic details, see: Bugaris & Ibers (2008 ▶) and Haneveld & Jellinek (1969 ▶). For stand­ard­ization of structural data, see: Gelato & Parthé (1987 ▶).

## Experimental

### 

#### Crystal data

UPSe
*M* *_r_* = 347.96
Tetragonal, 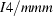

*a* = 3.9443 (4) Å
*c* = 16.2836 (17) Å
*V* = 253.33 (4) Å^3^
*Z* = 4
Mo *K*α radiation
μ = 78.66 mm^−1^
*T* = 298 K
0.41 × 0.39 × 0.03 mm

#### Data collection

Bruker APEXII CCD diffractometer
Absorption correction: numerical face-indexed (*SADABS*; Sheldrick, 2008*a* ▶) *T*_min_ = 0.013, *T*_max_ = 0.175
1506 measured reflections
128 independent reflections
128 reflections with *I* > 2σ(*I*)
*R*_int_ = 0.034

#### Refinement

*R*[*F* ^2^ > 2σ(*F* ^2^)] = 0.024
*wR*(*F* ^2^) = 0.052
*S* = 1.31
128 reflections
12 parameters
Δρ_max_ = 1.89 e Å^−3^
Δρ_min_ = −4.77 e Å^−3^

### 

Data collection: *APEX2* (Bruker, 2009 ▶); cell refinement: *SAINT* (Bruker, 2009 ▶); data reduction: *SAINT*; program(s) used to solve structure: *SHELXS97* (Sheldrick, 2008*b* ▶); program(s) used to refine structure: *SHELXL97* (Sheldrick, 2008*b* ▶); molecular graphics: *CrystalMaker* (Palmer, 2009 ▶); software used to prepare material for publication: *SHELXL97*.

## Supplementary Material

Crystal structure: contains datablock(s) I, global. DOI: 10.1107/S1600536811049592/wm2560sup1.cif

Structure factors: contains datablock(s) I. DOI: 10.1107/S1600536811049592/wm2560Isup2.hkl

Additional supplementary materials:  crystallographic information; 3D view; checkCIF report

## Figures and Tables

**Fig. 1.**
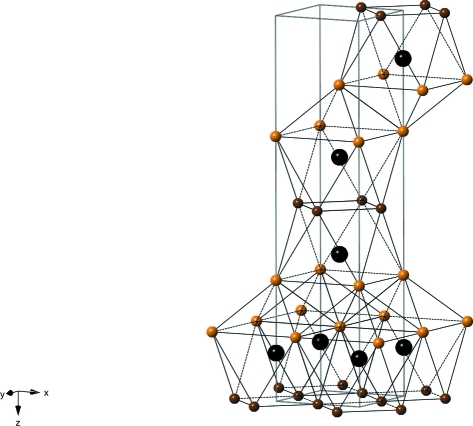
Structure of β-UPSe. U atoms are black, P atoms are brown, and Se atoms are orange.

**Fig. 2.**
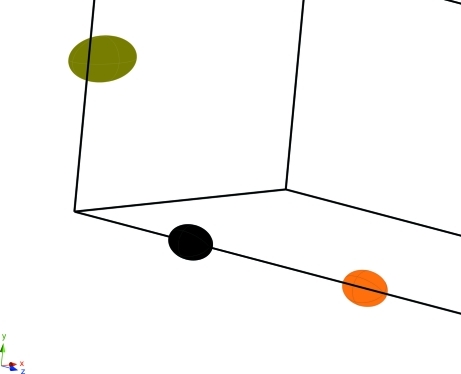
: Asymmetric unit of β-UPSe. Displacement ellipsoids are drawn at the 95% probability level. Color code as in Fig. 1.

## supplementary crystallographic information

### Comment

The compounds U*TQ* (*T* = P, As, As, Bi; *Q* = S, Se, Te) crystallize in one of two structure types. UBiTe, U*TQ* (*T* = As, Sb; *Q* = S, Se, Te), and UP*Q* (*Q* = S, Se) (Hulliger, 1968; Leciejewicz & Zygmunt, 1972; Haneveld & Jellinek, 1969; Zygmunt *et al.*, 1974*a*; Pietraszko & Lukaszewicz, 1975; Pearson, 1985) crystallize in the tetragonal space group *P*4/*nmm* in the PbFCl (Nieuwenkamp & Bijvoet, 1932) structure type. UPTe and UAsTe (Pietraszko & Lukaszewicz, 1975; Pearson, 1985; Zygmunt *et al.*, 1974*b*) crystallize in the tetragonal space group *I*4/*mmm* in the UGeTe (Haneveld & Jellinek, 1969) structure type. Magnetic studies have been performed on single crystals of β-UPSe crystallizing in the UGeTe structure type, but no structural data were reported. β-UPSe orders antiferromagnetically at low temperature (Troc, 1987; Kaczorowski *et al.*, 1995).

We have synthesized β-UPSe from a CsCl flux, in contrast to previous reported synthetic methods. This compound crystallizes with four formula units in the tetragonal space group *I*4/*mmm* (Figure 1) in the UGeTe structure type. The asymmetric unit comprises atoms U1 (site symmetry 4*mm*), Se1 (4*mm*), and P1 (*mmm*.) (Figure 2). U is coordinated in a monocapped square-antiprismatic arrangement, where the square face is formed by P atoms and the other five vertices are Se atoms. These moieties face-share along the four triangular faces formed by two Se atoms and one P atom with adjacent moieties. They also face-share along the square faces formed by P atoms. Finally, each of the four edges on the cap are shared with another moiety so that the U atoms are staggered when viewed down [001]. Viewed on the side of the basal plane, each atom type lies on its own plane.

The structure is related to that of α-UPSe (Zygmunt *et al.*, 1974*a*; Hulliger, 1968). The only difference is that instead of face-sharing along the square faces formed by the P atoms, the moieties edge-share with four other moieties, such that the U atoms are staggered in a checkerboard pattern when viewed down [001].

Interatomic distances are typical. The U–P distance is 2.8514 (7) Å, comparable to 2.91 Å in α-UPSe (Zygmunt *et al.*, 1974*a*). The U–Se distances are 2.9605 (5) Å and 3.0633 (11) Å, compared to 2.901 Å and 3.131 Å in α-UPSe.

### Experimental

U filings (Oak Ridge National Laboratory) were powdered as previously described (Bugaris & Ibers, 2008; Haneveld & Jellinek, 1969), and U_2_Se_3_ was synthesized by the stoichiometric reaction of U and Se (Cerac 99.999%) in a fused-silica tube at 1273 K. Black square plates of β-UPSe were synthesized in the reaction of U_2_Se_3_ (0.215 mmol), red P (Aldrich 99%, 0.064 mmol), Se (0.215 mmol), and CsCl (MP Biomedical 99.9%, 1.21 mmol) in a carbon-coated fused-silica tube. Reagents were loaded in an argon-filled glove box, and the tube was flame-sealed under 10^—4^ Torr vacuum. The tube was placed in a computer-controlled furnace and heated to 1273 K in 96 h, held there for 4 h, cooled to 1223 K in 12 h, held there for 96 h, and then cooled to 298 K in 350 h. The solidified flux was washed off with water. Qualitative EDS analysis using a Hitachi S-3400 SEM showed the presence of U, P, and Se, with no detectable Cs or Cl content in the crystals.

### Refinement

The structure was standardized with the use of *STRUCTURE TIDY* (Gelato & Parthé, 1987). The highest peak in the difference electron density map is 1.9 (6) e/Å^3^, 0.03 Å from atom U1, and the deepest hole is -4.8 (6) e/Å^3^, 0.91 Å from atom U1.

The refinement results in the placement of a half-occupied P1 atom in the 8*j* position. The resultant disorder at +*x*,1/2,0 (P1a) and -*x*,1/2,0 (P1b) leads to impossible P1a–P1b distances of 0.52 Å and 2.42 Å. The P1 atoms are arranged in a square net 2.813 Å on a side. The distribution of P1a squares and P1b squares within a structure is random, as no supercell reflections were observed.

### Crystal data

**Table:**

UPSe	*D*_x_ = 9.123 Mg m^−^^3^
*M**_r_* = 347.96	Mo *K*α radiation, λ = 0.71073 Å
Tetragonal, *I*4/*m**m**m*	Cell parameters from 1519 reflections
Hall symbol: -I 4 2	θ = 5.0–28.4°
*a* = 3.9443 (4) Å	µ = 78.66 mm^−^^1^
*c* = 16.2836 (17) Å	*T* = 298 K
*V* = 253.33 (4) Å^3^	Square plate, black
*Z* = 4	0.41 × 0.39 × 0.03 mm
*F*(000) = 564	

### Data collection

**Table:**

Bruker APEXII CCD diffractometer	128 independent reflections
Radiation source: fine-focus sealed tube	128 reflections with *I* > 2σ(*I*)
graphite	*R*_int_ = 0.034
φ and ω scans	θ_max_ = 28.4°, θ_min_ = 2.5°
Absorption correction: numerical face-indexed (*SADABS*; Sheldrick, 2008*b*)	*h* = −5→5
*T*_min_ = 0.013, *T*_max_ = 0.175	*k* = −5→5
1506 measured reflections	*l* = −21→21

### Refinement

**Table:**

Refinement on *F*^2^	Primary atom site location: structure-invariant direct methods
Least-squares matrix: full	Secondary atom site location: difference Fourier map
*R*[*F*^2^ > 2σ(*F*^2^)] = 0.024	[1.00000 + 0.00000exp(0.00(sinθ/λ)^2^)]/ [σ^2^(*F*_o_^2^) + 0.0000 + 0.0000**P* + (0.0377*P*)^2^ + 0.0000sinθ/λ] where *P* = 1.00000*F*_o_^2^ + 0.00000*F*_c_^2^
*wR*(*F*^2^) = 0.052	(Δ/σ)_max_ < 0.001
*S* = 1.31	Δρ_max_ = 1.89 e Å^−^^3^
128 reflections	Δρ_min_ = −4.77 e Å^−^^3^
12 parameters	Extinction correction: *SHELXL97* (Sheldrick, 2008a), Fc^*^=kFc[1+0.001xFc^2^λ^3^/sin(2θ)]^-1/4^
0 restraints	Extinction coefficient: 0.0139 (12)

### Special details

**Table:**

Geometry. All e.s.d.'s (except the e.s.d. in the dihedral angle between two l.s. planes) are estimated using the full covariance matrix. The cell e.s.d.'s are taken into account individually in the estimation of e.s.d.'s in distances, angles and torsion angles; correlations between e.s.d.'s in cell parameters are only used when they are defined by crystal symmetry. An approximate (isotropic) treatment of cell e.s.d.'s is used for estimating e.s.d.'s involving l.s. planes.

### Fractional atomic coordinates and isotropic or equivalent isotropic displacement parameters (Å^2^)

**Table:**

	*x*	*y*	*z*	*U*_iso_*/*U*_eq_	Occ. (<1)
U1	0.0000	0.0000	0.125455 (18)	0.0090 (3)	
Se1	0.0000	0.0000	0.31358 (6)	0.0093 (4)	
P1	0.0660 (18)	0.5000	0.0000	0.021 (2)	0.50

### Atomic displacement parameters (Å^2^)

**Table:**

	*U*^11^	*U*^22^	*U*^33^	*U*^12^	*U*^13^	*U*^23^
U1	0.0066 (3)	0.0066 (3)	0.0138 (4)	0.000	0.000	0.000
Se1	0.0068 (4)	0.0068 (4)	0.0142 (6)	0.000	0.000	0.000
P1	0.040 (7)	0.0114 (18)	0.0107 (13)	0.000	0.000	0.000

### Geometric parameters (Å, °)

**Table:**

U1—P1^i^	2.8514 (7)	U1—U1^iv^	3.9443 (4)
U1—P1^ii^	2.8514 (7)	U1—U1^ii^	4.0858 (7)
U1—P1^iii^	2.8514 (7)	P1—P1^vii^	0.520 (14)
U1—P1^iv^	2.8514 (7)	P1—P1^xv^	2.421 (10)
U1—P1^v^	2.8514 (7)	P1—P1^vi^	2.421 (10)
U1—P1^vi^	2.8514 (7)	P1—P1^xvi^	2.8132 (13)
U1—P1^vii^	2.8514 (7)	P1—P1^iii^	2.8132 (13)
U1—P1	2.8514 (7)	P1—P1^xvii^	2.8132 (13)
U1—Se1^viii^	2.9605 (5)	P1—P1^v^	2.8132 (13)
U1—Se1^ix^	2.9605 (5)	P1—U1^ii^	2.8514 (7)
U1—Se1^x^	2.9605 (5)	P1—U1^xiv^	2.8514 (7)
U1—Se1^xi^	2.9605 (5)	P1—U1^vii^	2.8514 (7)
U1—Se1	3.0633 (11)	P1—P1^i^	3.157 (10)
U1—U1^xii^	3.9443 (4)	P1—P1^xviii^	3.157 (10)
U1—U1^xiii^	3.9443 (4)	P1—P1^xix^	3.424 (14)
U1—U1^xiv^	3.9443 (4)		
			
P1^i^—U1—P1^ii^	50.2 (2)	P1^iii^—U1—Se1^xi^	139.62 (13)
P1^ii^—U1—P1^iii^	59.116 (17)	P1^iv^—U1—Se1^xi^	73.66 (10)
P1^i^—U1—P1^iv^	59.116 (17)	P1^v^—U1—Se1^xi^	73.66 (10)
P1^iii^—U1—P1^iv^	67.2 (2)	P1^vi^—U1—Se1^xi^	80.81 (10)
P1^i^—U1—P1^v^	87.52 (3)	P1^vii^—U1—Se1^xi^	139.62 (13)
P1^ii^—U1—P1^v^	59.116 (17)	P1—U1—Se1^xi^	129.81 (13)
P1^iii^—U1—P1^v^	88.48 (3)	Se1^viii^—U1—Se1^xi^	83.543 (13)
P1^iv^—U1—P1^v^	50.2 (2)	Se1^ix^—U1—Se1^xi^	83.543 (13)
P1^i^—U1—P1^vi^	88.48 (3)	Se1^x^—U1—Se1^xi^	140.81 (4)
P1^ii^—U1—P1^vi^	67.2 (2)	P1^i^—U1—Se1	135.762 (15)
P1^iii^—U1—P1^vi^	87.52 (3)	P1^ii^—U1—Se1	135.762 (15)
P1^iv^—U1—P1^vi^	59.116 (17)	P1^iii^—U1—Se1	135.762 (15)
P1^i^—U1—P1^vii^	59.116 (17)	P1^iv^—U1—Se1	135.762 (15)
P1^ii^—U1—P1^vii^	87.52 (3)	P1^v^—U1—Se1	135.762 (15)
P1^iii^—U1—P1^vii^	50.2 (2)	P1^vi^—U1—Se1	135.762 (15)
P1^iv^—U1—P1^vii^	88.48 (3)	P1^vii^—U1—Se1	135.762 (15)
P1^v^—U1—P1^vii^	67.2 (2)	P1—U1—Se1	135.762 (15)
P1^vi^—U1—P1^vii^	59.116 (17)	Se1^viii^—U1—Se1	70.41 (2)
P1^i^—U1—P1	67.2 (2)	Se1^ix^—U1—Se1	70.41 (2)
P1^ii^—U1—P1	88.48 (3)	Se1^x^—U1—Se1	70.41 (2)
P1^iii^—U1—P1	59.116 (17)	Se1^xi^—U1—Se1	70.41 (2)
P1^iv^—U1—P1	87.52 (3)	P1^i^—U1—P1^xx^	47.14 (19)
P1^v^—U1—P1	59.116 (17)	P1^ii^—U1—P1^xx^	25.69 (12)
P1^vi^—U1—P1	50.2 (2)	P1^iii^—U1—P1^xx^	57.61 (9)
P1^i^—U1—Se1^viii^	139.62 (13)	P1^iv^—U1—P1^xx^	34.53 (13)
P1^ii^—U1—Se1^viii^	139.62 (13)	P1^v^—U1—P1^xx^	84.63 (11)
P1^iii^—U1—Se1^viii^	129.81 (13)	P1^vi^—U1—P1^xx^	92.92 (11)
P1^iv^—U1—Se1^viii^	129.81 (13)	P1^vii^—U1—P1^xx^	101.21 (11)
P1^v^—U1—Se1^viii^	80.81 (10)	P1—U1—P1^xx^	105.774 (19)
P1^vi^—U1—Se1^viii^	73.66 (10)	Se1^viii^—U1—P1^xx^	163.21 (3)
P1^vii^—U1—Se1^viii^	80.81 (10)	Se1^ix^—U1—P1^xx^	48.49 (3)
P1—U1—Se1^viii^	73.66 (10)	Se1^x^—U1—P1^xx^	113.11 (3)
P1^i^—U1—Se1^ix^	73.66 (10)	Se1^xi^—U1—P1^xx^	84.38 (5)
P1^ii^—U1—Se1^ix^	73.66 (10)	Se1—U1—P1^xx^	116.05 (3)
P1^iii^—U1—Se1^ix^	80.81 (10)	Se1^viii^—U1—P1^xxi^	163.21 (3)
P1^iv^—U1—Se1^ix^	80.81 (10)	Se1^ix^—U1—P1^xxi^	48.49 (2)
P1^v^—U1—Se1^ix^	129.81 (13)	Se1^x^—U1—P1^xxi^	84.38 (5)
P1^vi^—U1—Se1^ix^	139.62 (13)	Se1^xi^—U1—P1^xxi^	113.11 (3)
P1^vii^—U1—Se1^ix^	129.81 (13)	Se1—U1—P1^xxi^	116.05 (3)
P1—U1—Se1^ix^	139.62 (13)	U1^viii^—Se1—U1^ix^	140.82 (4)
Se1^viii^—U1—Se1^ix^	140.81 (4)	U1^viii^—Se1—U1^x^	83.544 (13)
P1^i^—U1—Se1^x^	80.81 (10)	U1^ix^—Se1—U1^x^	83.544 (13)
P1^ii^—U1—Se1^x^	129.81 (13)	U1^viii^—Se1—U1^xi^	83.544 (13)
P1^iii^—U1—Se1^x^	73.66 (10)	U1^ix^—Se1—U1^xi^	83.544 (13)
P1^iv^—U1—Se1^x^	139.62 (13)	U1^x^—Se1—U1^xi^	140.82 (4)
P1^v^—U1—Se1^x^	139.62 (13)	U1^viii^—Se1—U1	109.59 (2)
P1^vi^—U1—Se1^x^	129.81 (13)	U1^ix^—Se1—U1	109.59 (2)
P1^vii^—U1—Se1^x^	73.66 (10)	U1^x^—Se1—U1	109.59 (2)
P1—U1—Se1^x^	80.81 (10)	U1^xi^—Se1—U1	109.59 (2)
Se1^viii^—U1—Se1^x^	83.543 (13)	U1^xxii^—P1—U1^xxiii^	176.41 (10)
Se1^ix^—U1—Se1^x^	83.543 (13)	U1^xii^—P1—U1^xxiii^	131.945 (10)
P1^i^—U1—Se1^xi^	129.81 (13)	U1^xix^—P1—U1^xxiii^	130.116 (13)
P1^ii^—U1—Se1^xi^	80.81 (10)	U1^xxiv^—P1—U1^xxiii^	108.344 (10)

Symmetry codes: (i) *y*−1, −*x*, −*z*; (ii) −*x*, −*y*, −*z*; (iii) −*y*, *x*, *z*; (iv) *x*, *y*−1, *z*; (v) *y*, −*x*, −*z*; (vi) −*y*+1, *x*, *z*; (vii) −*x*, −*y*+1, −*z*; (viii) −*x*+1/2, −*y*+1/2, −*z*+1/2; (ix) −*x*−1/2, −*y*−1/2, −*z*+1/2; (x) −*x*−1/2, −*y*+1/2, −*z*+1/2; (xi) −*x*+1/2, −*y*−1/2, −*z*+1/2; (xii) *x*+1, *y*, *z*; (xiii) *x*−1, *y*, *z*; (xiv) *x*, *y*+1, *z*; (xv) *y*, −*x*+1, −*z*; (xvi) −*y*+1, *x*+1, *z*; (xvii) *y*−1, −*x*+1, −*z*; (xviii) −*y*, *x*+1, *z*; (xix) −*x*+1, −*y*+1, −*z*; (xx) −*y*, *x*−1, *z*; (xxi) *x*−1, *y*−1, *z*; (xxii) *x*+1, *y*+1, *z*; (xxiii) −*x*−1, −*y*, −*z*; (xxiv) −*x*+1, −*y*, −*z*.
